# Supplementary material for: Development and Validation of the Values Internalization Scale
Source: Behav Sci (Basel). 2025 May 12;15(5):660. doi: 10.3390/bs15050660 (PMC12109008; doi:10.3390/bs15050660)
Supplement: Supplementary file 1 [file behavsci-15-00660-s001.zip › behavsci-3572190-supplementary.pdf]

The Values Internalization Scale (VIS)

English version of VIS

Instructions: We welcome your participation in this questionnaire. Please consider the following statement : "**（Fill the values to be measured in here）.**" To what extent does this statement align with your reality? Please respond to the subsequent questions by selecting the corresponding number from the provided options: 1 = Strongly Disagree; 2 = Disagree; 3 = Uncertain; 4 = Agree; 5 = Strongly Agree. It is important to note that everyone holds unique values, so there are no right or wrong answers. We encourage you to respond as accurately and truthfully as possible."

Item Number	Dimension	Items	Strongly Disagree	Disagree	Uncertain	Agree	Strongly Agree
1	Ignoring-resisting stage	I reject this value.	1	2	3	4	5
2		I will not follow this value even if there is a reward.	1	2	3	4	5
3		I don't understand this value.	1	2	3	4	5
4		My actions are contrary to this value.	1	2	3	4	5
5		Even if ordered by others, I am unwilling to act according to this value.	1	2	3	4	5
6		I would not consider practicing this value.	1	2	3	4	5
7		This value is remote from my life.	1	2	3	4	5
8	Understanding stage	This value is meaningful, but I have not yet applied it to my life.	1	2	3	4	5
9		Although I value this belief, I have not practiced it.	1	2	3	4	5
10		This value is important, but I do not adjust my behavior based on it.	1	2	3	4	5
11		This value is important, yet I do not adhere to it as a personal standard.	1	2	3	4	5
12		I often forget that my behavior should align with this value.	1	2	3	4	5

13		I don't have a clear understanding of this value.	1	2	3	4	5
14	Attempting to practice stage	I practice this value to maintain relationships with others.	1	2	3	4	5
15		I try to put this value into action to gain profit.	1	2	3	4	5
16		I act on this value to avoid punishment.	1	2	3	4	5
17		I start practicing this value to gain others' approval or appreciation.	1	2	3	4	5
18		I attempt to act on this value due to external supervision.	1	2	3	4	5
19	Integration stage	This value is more important than anything else.	1	2	3	4	5
20		This value is the foundation for other things.	1	2	3	4	5
21		When I violate this value, I engage in compensatory behaviors.	1	2	3	4	5
22		I take the initiative to spread values to others.	1	2	3	4	5
23		I have negative feelings when I violate these values.	1	2	3	4	5
24		I will not change this value when facing external temptations.	1	2	3	4	5
25		I have a deep understanding of this value.	1	2	3	4	5

Chinese version of VIS

指导语：欢迎参加此次问卷调查。请你根据："（此处填入需要测量的价值观）"。这一描述与你自身实际情况相符合的程度，对下列题项进行回答，并在所提供的选项和相应的数字上打 √。1=非常不同意；2=不同意；3=不确定；4=同意；5=非常同意。每个人的价值观各不相同，所以答案没有对错之分，请你尽可能准确和真实地作答。

题目序号	维度	项目	非常不同意	不同意	不确定	同意	非常同意
1	忽视-抗拒期	我抵触这个价值观。	1	2	3	4	5
2		即使有奖励，我也不会遵守这个价值观。	1	2	3	4	5
3		我不理解这个价值观。	1	2	3	4	5
4		我的行为与这个价值观相反。	1	2	3	4	5
5		即使别人命令我，我也不愿意按照这个价值观行事。	1	2	3	4	5
6		我不会考虑践行这个价值观。	1	2	3	4	5
7		这个价值观与我的生活距离遥远。	1	2	3	4	5
8	理解期	这个价值观很有意义，但我还没有把它应用到我的生活中。	1	2	3	4	5
9		尽管我重视这个价值观，但我并没有真正去实践它。	1	2	3	4	5
10		这个价值观重要，但我不会根据它调整我的行为。	1	2	3	4	5
11		这个价值观很重要，但我不会用它来要求自己。	1	2	3	4	5
12		我常常会忘记我的行为要遵循这个价值观。	1	2	3	4	5
13		我对这个价值观的认知不够清晰。	1	2	3	4	5
14	尝试践行期	我为了维持与他人的关系，而践行这个价值观。	1	2	3	4	5
15		我为了获得利益，尝试将这个价值观付诸行动。	1	2	3	4	5
16		为了避免惩罚，我按照这个价值观行事。	1	2	3	4	5
17		为了获得他人的赞同/赞赏，我开始践行这个价值观。	1	2	3	4	5
18		我因为外部监督，尝试将这个价值观付诸行动。	1	2	3	4	5
19		价值观比其他事物更重要。	1	2	3	4	5
20		这个价值观是其他事物的基础。	1	2	3	4	5

21	融合期	违背这个价值观后我会产生弥补行为。	1	2	3	4	5
22		我主动向他人传播价值观。	1	2	3	4	5
23		违背这个价值观我会产生消极情绪。	1	2	3	4	5
24		面对外界诱惑我也不会改变这个价值观。	1	2	3	4	5
25		我对这个价值观的理解深刻。	1	2	3	4	5
